# Anomalous origin of left coronary arteries from the pulmonary artery in an asymptomatic adult: An autopsy case report

**DOI:** 10.1097/MD.0000000000017212

**Published:** 2019-09-20

**Authors:** Majd Al Shaarani, Yasir Alzubaidi, Long Jin

**Affiliations:** Department of Pathology and Translational Pathobiology, Louisiana State University Health Science Center in Shreveport, Shreveport, LA.

**Keywords:** ALCAPA, anomalous origin of the left coronary artery from the pulmonary artery, coronary artery anomalies, high take-off

## Abstract

**Rationale:**

Anomalous Origin of Left Coronary Arteries from the Pulmonary Artery (ALCAPA), also known as Bland-White-Garland (BWG), is a rare form of coronary artery anomaly that is usually discovered in the first few months of life. Only rarely can patients with this anomaly reach adulthood without symptoms.

**Patient concerns:**

A 28-year-old female was witnessed suddenly collapse with a seizure-like episode by her colleagues at work.

**Diagnosis and Intervention:**

Routine cardiopulmonary resuscitation was performed by emergency medical service technologists. The patient was unable to be revived. Postmortem examination revealed the patient had ALCAPA with a focal chronic ischemic injury of the left ventricle. Moreover, a high take-off of the right coronary artery was also discovered.

**Outcomes:**

The patient passed away due to ALCAPA. The mechanism of death was cardiac arrhythmia being triggered by myocardial ischemic changes.

**Lessons:**

In the rare cases where ALCAPA manifests in an asymptomatic adult, the mortality rate is very high. This case demonstrates the importance of awareness of such patients living under the tremendous risk of sudden cardiac death.

## 1 Introduction

Coronary artery anomalies are rare and affect about 1% of the general population. The chance of finding these anomalies in autopsies is very rare.^[1]^ Anomalous origin of the left coronary artery from the pulmonary artery (ALCAPA, also known as Bland-White-Garland [BWG]) is a rare form of congenital coronary artery anomaly with an incidence of about 1 in every 300,000 live births or 0.25% to 0.5% of patients with congenital heart disease.^[2–6]^ In patients with ALCAPA syndromes, only 10% survive beyond childhood.^[3]^ The occurrence of this anomaly is not confined to the pediatric population as it can, in sporadic cases, manifest in adults with catastrophic presentation as in this autopsy. When clinically asymptomatic, these anomalies are discovered incidentally during the angiogram, a computed tomography (CT) or echocardiography.^[3,7,8]^

## 2 Case report

An Institutional Review Board (IRB) waiver of patient consent was obtained for the publication of this case report.

**Figure 1:**
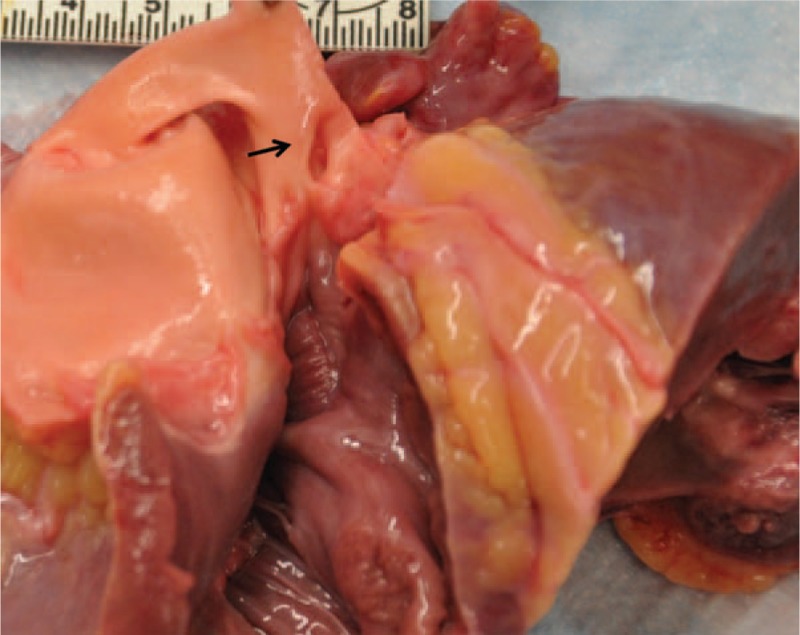
The LCA is originating from the pulmonary artery (arrow). LCA = Left Coronary Artery.

**Figure 2:**
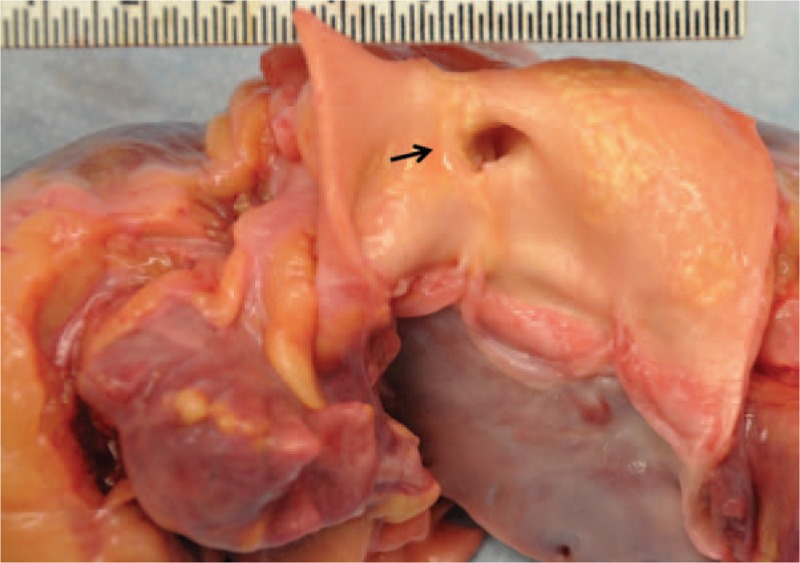
The RCA is originating from the aorta with a high take-off point (arrow). RCA = Right Coronary Artery.

**Figure 3:**
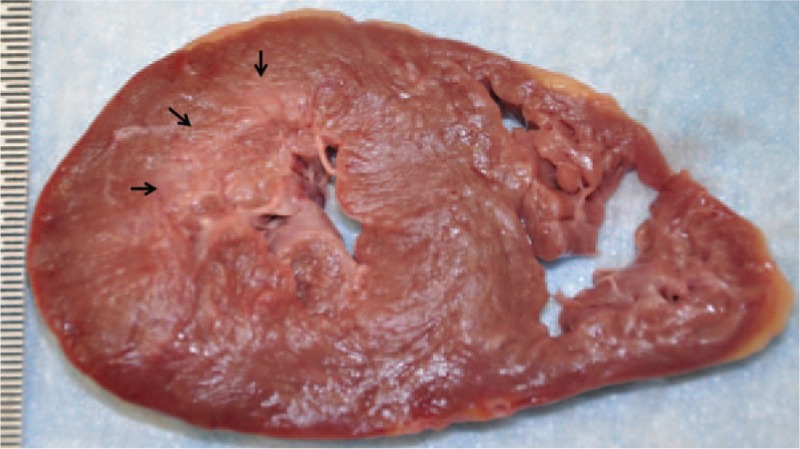
The left ventricle with subendocardial fibrosis at the anterolateral aspect to include the papillary muscle (arrows).

**Figure 4:**
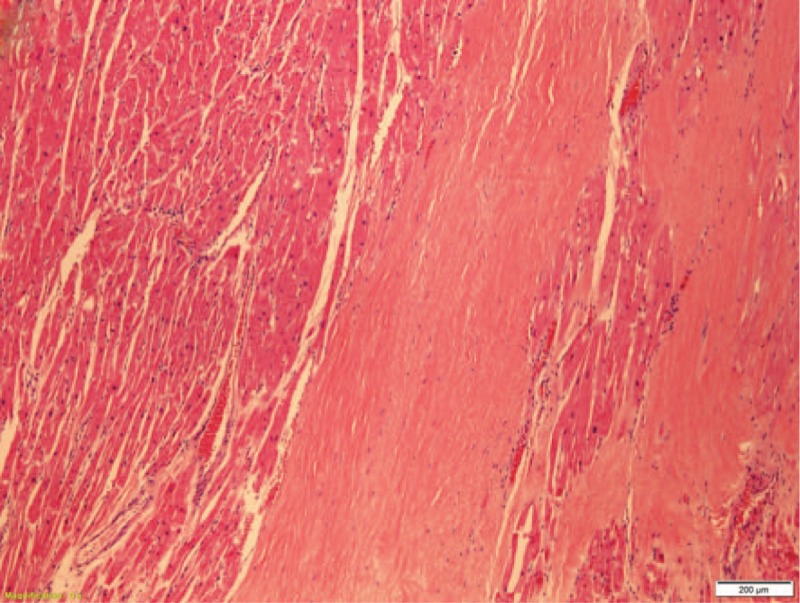
Scattered fibrosis in the papillary muscle (40x).

**Figure 5:**
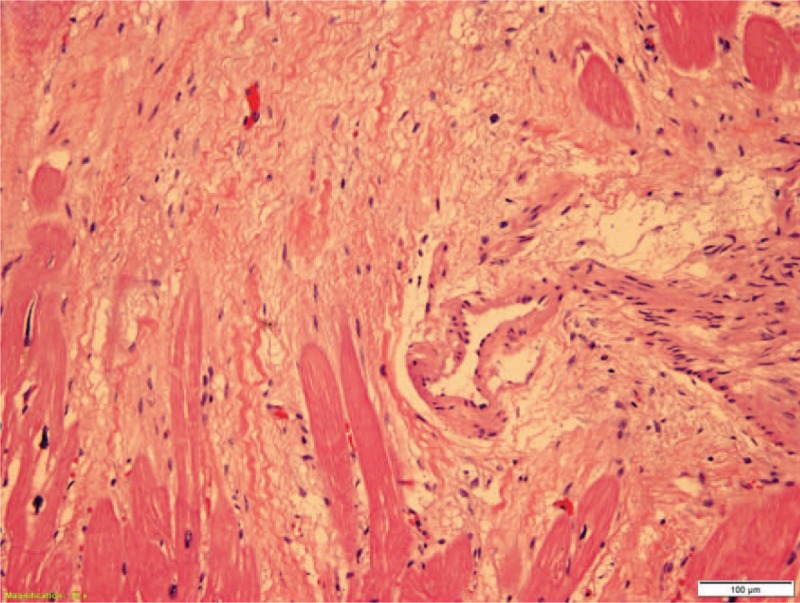
The anterolateral aspect of the left ventricle (100x).

**Figure 6:**
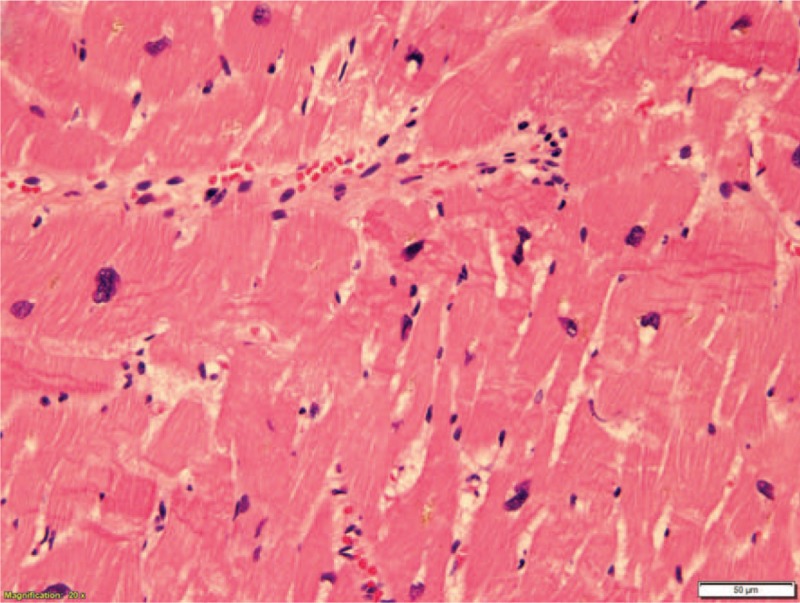
Contraction band necrosis (200x).

**Figure 7:**
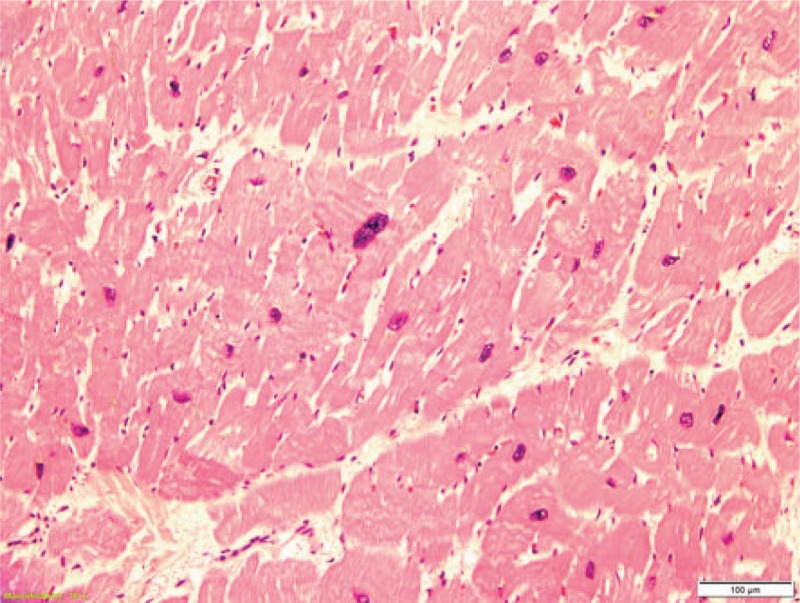
Hypertrophic cardiomyocytes with boxcar shaped nuclei (100x).

A 28-year-old African American female suddenly collapsed at work. The co-workers who witnessed the incident described her as having a seizure-like episode. No past medical history was available to us, but apparently the deceased was healthy and asymptomatic. In autopsy, except for coronary artery abnormalities, the external and internal examinations were mostly unremarkable. The heart was 370 grams. Transverse and longitudinal dissections of the coronary artery system revealed an ALCAPA, Figure 1. The left coronary ostium was located in the pulmonary root. The lumina of the left anterior descending and left circumflex coronary arteries were significantly small. A single coronary ostium was present in the aortic root, and its location was much higher than normal (10 mm above the sinotubular junction, Fig. 2). The right coronary artery appeared tortuous and dilated during its course, especially the proximal portion. On opening the heart, the myocardium of the left ventricle was 1.3 cm in thickness. The right ventricular wall was 0.3 cm in thickness. Focal fibrosis was present at the anterolateral free wall of the left ventricle, measuring 2 cm in the largest dimension, Figure 3. The valve surfaces were smooth, opaque, and glistening. The tricuspid, pulmonic, mitral, and aortic valves were 9.8 cm, 5.7 cm, 9.9 cm, and 6.0 cm, respectively. All chambers were of normal configuration. The intimal surface of the aorta was free of atherosclerosis. Microscopic examination of multiple sections of the cardiac tissue revealed patchy myocardial fibrosis prominent at the papillary muscle and subendocardium, corresponding with remote myocardial ischemic injury, Figures 4 and 5. The sections also revealed subtle changes consistent with acute ischemic injury, including myocyte hypereosinophilia, a few interstitial neutrophils, shrunken pyknotic nuclei and contraction band necrosis, Figure 6. Additionally, there was arteriosclerosis and areas of hypertrophic cardiomyocytes with boxcar shaped nuclei, Figure 7.

The cause of death was determined to be the ALCAPA. The mechanism of death was cardiac arrhythmia due to myocardial ischemic injuries.

## 3 Discussion

ALCAPA, also known as BWG is a rare form of coronary artery anomaly with an incidence of about 1 in every 300,000 live births or 0.25% to 0.5% of patients with congenital heart disease.^[2–6]^ The occurrence, however, might be underestimated since many patients may be asymptomatic.^[4]^ Without treatment, approximately 90% of infants die within the first year of life.^[11]^ It is usually an isolated anomaly, but the association with other anomalies such as atrial/ventricular septal defect, patent ductus arteriosus, coarctation of the aorta, and tetralogy of Fallot have been reported.^[5,6]^ In the present case, besides ALCAPA, high take-off, the right coronary artery is also identified.

The pathophysiology and hence, the clinical presentation go through 3 phases.^[3]^ Initially, due to the persistent high pulmonary artery pressure, blood from the pulmonary artery flows into the anomalous left coronary artery is maintained, and the infant is asymptomatic. During the next phase, with the closure of the ductus arteriosus and the decreasing pulmonary artery pressure, the blood flow through the anomalous artery becomes increasingly compromised and eventually becomes retrograde. In the last phase, the collaterals developed between the right coronary artery (RCA) and the anomalous left coronary artery (LCA) play an essential role in survival in this phase. If these collaterals are large enough, blood shunts from RCA to LCA, and in a retrograde fashion back into the pulmonary artery root. This flow keeps the patient alive but is often insufficient, and the chronically hypo-perfused left ventricle becomes ischemic especially in the subendothelial area.^[2–6]^ About 15% of the patient has enough collaterals to survive this phase.^[9]^ Patients may present with a variety of symptoms ranging from asymptomatic, respiratory infections, palpitations, fatigue, angina, ischemic cardiomyopathy, congestive heart failure, dysrhythmia, and sudden cardiac death.^[1,2,4–6,10]^ The papillary muscle of the mitral valve is also especially susceptible, and symptoms of mitral regurgitation with an audible murmur may predominate the presentation.^[10,11]^ There is always left ventricular hypertrophy and scattered fibrosis.^[9,10]^

In a review of 151 cases of ALCAPA in adults, the average reported age was 41 years. 66% of the patients presented symptoms of angina, fatigue, dyspnea, or palpitation. 17% of the patients suffered from syncope, ventricular arrhythmia, or sudden death. 14% of the patients (a total of 21 cases) were asymptomatic. 12% of the patients (total 18 cases) were diagnosed in autopsy with the average age of sudden cardiac death being 31 years old ± 11.^[11]^

Although angiogram is the gold standard for the diagnosis of this anomaly, other diagnostic modalities (cardiac CT and magnetic resonance imaging (MRI), echocardiography, and even electrocardiography (ECG)) may be helpful and even diagnostic.^[2,4,7,11]^ Surgery, with the idea of creating a 2-artery coronary system, is the definitive treatment. Several surgical techniques and options are available with most direct and advisable one being the translocation of the LCA into the aortic root.^[10]^ It has been well-documented that the surgical procedure using the 2-artery coronary system reached lower mortality (0%–14%) since 1995.^[11]^

## 4 Conclusion

ALCAPA is not confined to pediatric populations and can manifest in adults. A fair number of these patients are diagnosed in an autopsy. Therefore, the awareness of the existence of such anomaly is essential in increasing patients’ survival. Attention should be paid when examining the heart in a young adult patient with sudden death.

## Author contributions

**Validation:** Long Jin.

**Writing – original draft:** Majd Al Shaarani, Yasir AlZubaidi.

**Writing – review & editing:** Majd Al Shaarani, Long Jin.

Majd Al Shaarani orcid: 0000-0003-0295-5276.
